# “I could have a proper ankle” – a qualitative study of patients’ perceptions of total ankle replacement and ankle fusion surgery

**DOI:** 10.1186/s13047-022-00595-8

**Published:** 2022-12-12

**Authors:** Anna M. Anderson, Lara S. Chapman, Heidi J. Siddle, Sue Watson, Jane Klugerman, Deborah Antcliff, Anne-Maree Keenan, Claire L. Brockett

**Affiliations:** 1 Leeds Institute of Rheumatic & Musculoskeletal Medicine, University of Leeds, Leeds, UK; 2 School of Healthcare, University of Leeds, Leeds, UK; 3 Leeds Institute of Health Sciences, University of Leeds, Leeds, UK; 4 Leeds National Institute for Health and Care Research Biomedical Research Centre, Leeds, UK; 5 Bury Integrated Pain Service, Bury Care Organisation, Northern Care Alliance NHS Foundation Trust, Bury, England, UK; 6 School of Medicine, Keele University, Keele, UK; 7 Institute of Medical and Biological Engineering, School of Mechanical Engineering, University of Leeds, Leeds, UK

**Keywords:** Total ankle replacement, Ankle fusion, Patient education, Rehabilitation

## Abstract

**Background:**

End-stage ankle osteoarthritis typically causes severe pain and impaired function. Surgical treatment involves total ankle replacement (TAR) or ankle fusion. Definitive evidence about which procedure is optimal is lacking. No previous studies have thoroughly explored patients’ experiences across the entire TAR/ankle fusion pathway. This study aimed to address this gap by exploring perceptions of surgery, education, rehabilitation and outcomes among patients who had undergone TAR or ankle fusion.

**Methods:**

Seven participants were purposively selected from an orthopaedic centre in northern England (3 females, 4 males). Participants had undergone primary TAR without revision (*n* = 2), TAR requiring revision (*n* = 3) or ankle fusion (*n* = 2). Each participant completed a single semi-structured interview. Interviews were digitally recorded, transcribed verbatim and analysed thematically.

**Results:**

Three themes, each with two subthemes, were identified: decision-making (seeking help; surgical options), perceptions of support (information/education; clinical support) and impact on the individual (personal circumstances and beliefs; post-operative outcomes). Pain affecting participants’ valued activities was key to their decision to seek help. Participants’ decision between TAR and ankle fusion was influenced by multiple factors. Concerns regarding the lack of joint flexibility following fusion were highlighted, with some participants perceiving TAR as a *“proper ankle”* that would enable them to avoid limping. Participants obtained information from various sources, with most feeling that the education from their care team was inadequate. Participants’ individual circumstances and beliefs influenced their decision-making and perceptions of their post-operative outcomes. Finally, whilst most participants were pleased with their outcomes, some experienced substantial ongoing problems such as difficulty walking and chronic pain.

**Conclusions:**

This study demonstrates the importance of providing adequate education about TAR and ankle fusion to enable patients to make informed decisions. Most participants felt that the education and clinical support they received did not fully meet their needs. Participants’ personal circumstances and beliefs had a strong influence on their decision-making and perceptions of their post-operative outcomes, highlighting the need to personally tailor education and clinical support. Future work with a larger sample of patients and other key stakeholders is required to develop consensus-based guidelines on pre- and post-operative support for patients undergoing TAR/ankle fusion.

**Supplementary Information:**

The online version contains supplementary material available at 10.1186/s13047-022-00595-8.

## Background

End-stage ankle osteoarthritis (OA) is a highly debilitating condition associated with severe pain, impaired function and reduced quality of life [1, 2]. The burden of ankle OA is substantial; over 29,000 patients in the United Kingdom (UK) are referred for a surgical opinion each year [3]. The main surgical treatment options for ankle OA are total ankle replacement (TAR) and ankle fusion (arthrodesis). The superiority of TAR versus ankle fusion remains controversial [4, 5]. The first prospective randomised controlled trial (RCT) directly comparing these treatments is underway in the UK [6].

Previous research suggests patients are influenced by a variety of sources, including their surgeon, peers and the Internet, when deciding between TAR and ankle fusion [7]. Patients living with both a TAR and ankle fusion have indicated a preference for their TAR due to maintenance of joint flexibility [8]. Correspondingly, a qualitative study exploring barriers to recruitment to the ongoing RCT comparing TAR and ankle fusion identified that most patients approached about the trial wanted to undergo TAR rather than ankle fusion [9]. However, another study highlighted that patients were concerned about balance, stability and potential damage to their ankle regardless of the type of surgery they had received [10]. These studies have increased our understanding of a limited number of aspects of patients’ perspectives of TAR and ankle fusion. However, patients’ lived experiences across the entire care pathway from the decision to seek advice for a painful ankle through to post-operative recovery have not been thoroughly explored.

The aim of this study was to help address an identified gap in practice knowledge by exploring perceptions of surgery, education, rehabilitation and outcomes among patients who have undergone TAR or ankle fusion, to inform clinical practice and future research.

## Methods

### Study design and reporting

We employed a qualitative research design to gain an in-depth understanding of patients’ perceptions of TAR and ankle fusion surgery. The study was informed by phenomenology as we sought to explore the meaning and shared understanding of participants’ lived experiences [11]. The study is reported in line with the consolidated criteria for reporting qualitative studies (COREQ) framework [12].

### Ethical approval

Ethical approval was obtained from the North West – Greater Manchester East Research Ethics Committee (18/NW/0819).

### Research team

Our research team included researchers with expertise in qualitative research, rheumatology, orthopaedics, podiatry, physiotherapy and biomechanical engineering, and two Patient and Public Involvement (PPI) contributors (SW, JK). Both PPI contributors have lived experience of being offered TAR and ankle fusion, with one having undergone TAR and the other opting to continue conservative management.

### Participants

Individuals who met the criteria specified in Table 1 were eligible to participate in the study. We purposively selected participants based on type of operation, length of time post-operatively and gender to maximise diversity of perspectives [11].

**Table 1:** Participant eligibility criteria

Inclusion criteria	Exclusion criteria
• Adult (aged 18 years old or over). • Undergone elective primary TAR or ankle fusion due to end-stage ankle arthritis at the recruitment site (with or without subsequent revision surgery). • At least 6 weeks post-operative. • Fluent in English.	• TAR or ankle fusion undertaken as an emergency procedure. • Unable to understand and speak English. • Major cognitive impairment or other impairment that would prevent engagement with the interview.

*TAR* total ankle replacement

Participants were recruited from a single orthopaedic centre within a teaching hospital in northern England. Potential participants were identified from clinic lists, approached in person at a routine clinic appointment or via an invitation letter and given the opportunity to discuss the study with a researcher at their clinic appointment or via telephone. All individuals who were screened and met the eligibility criteria agreed to participate in the study, resulting in a sample of seven people. This is in line with typical sample sizes for phenomenological studies, which focus on exploring the experiences of a relatively small number of participants in depth [11, 13].

### Data collection

Semi-structured interviews were conducted between 8th March 2019 and 12th March 2020 using a topic guide (Additional File 1), developed in conjunction with a PPI contributor (SW). Questions were open-ended and structured around the research aim. Each participant completed a single interview and provided written informed consent prior to their interview. One or two female researchers who were previously unknown to the participants, conducted each interview. When two researchers were present, one led the interview and the other mainly observed but was able to ask questions as appropriate. The interviewers included a physiotherapist working as a healthcare researcher (AMA), a biomechanical engineer with expertise in ankle joint interventions (CLB), and an experienced healthcare researcher (AMK). Interviews were conducted face-to-face in participants’ own homes (*n* = 4) or a hospital-based research centre (*n* = 3). One participant was accompanied by a family member during their interview. Interviews were digitally recorded, transcribed verbatim and supplemented with field notes. The interview duration ranged from 25 to 74 minutes.

### Data analysis

All interview transcripts were uploaded into NVivo 12 (QSR International; 2018) and analysed using reflexive thematic analysis [14, 15]. Initial coding was undertaken collaboratively by two researchers (AMA, CLB). Both researchers read and re-read the transcripts, collaboratively generated initial codes and collated similar codes together, holding regular discussions to review the coding. This was undertaken over a period of approximately eight months. The coding was inductive and focused on manifest content [14, 15]. A third researcher (DA) verified the coding for two randomly selected transcripts to assure credibility. Codes were grouped into provisional themes through a team discussion, then reviewed against the whole data set by two other researchers (HJS, LSC). Discrepancies in coding were settled by group consensus between all coders. Findings were discussed with all research team members, including our PPI contributors (SW, JK).

### Trustworthiness

We employed multiple strategies to address the trustworthiness criteria proposed by Lincoln and Guba [16], including:

- involving multiple researchers from different background in the analysis process and discussing the findings with our PPI contributors to enhance credibility;
- verifying the accuracy of the transcripts, employing reflexive thematic analysis, and providing quotes supporting our themes/subthemes to enhance confirmability;
- maintaining an audit trail and reporting detailed information about our study procedures to enhance dependability;
- purposively sampling participants and reporting detailed information about our study design, context, and participants to enhance transferability.

Member checking is another strategy that has been proposed to enhance credibility [16]. However, its appropriateness has been questioned due to the interpretative nature of qualitative data analysis and its potential to raise ethical issues [17, 18]. Due to these potential issues, and the fact that we discussed the findings with our PPI contributors, we did not incorporate member checking in the study design.

## Results

Table 2 presents the participant characteristics.

**Table 2:** Participant characteristics

Pseudonym	Gender	Age at interview	Type of surgery	Purposive selection category (time post-operatively)
Rose	Female	85	Right TAR	6 weeks – 6 months
Sarah	Female	77	Left ankle fusion	1–2 years
Edward	Male	72	Left TAR	Awaiting revision
George	Male	71	Right ankle fusion	6 weeks – 6 months
Mary	Female	66	Right TAR and revision to fusion	Post TAR revision
Henry	Male	72	Right TAR	> 5 years
Jack	Male	74	Left TAR and revision to fusion^a^	Post TAR revision

*TAR* total ankle replacement

^a^ Also had a right TAR approximately 3 years prior to the interview

**Fig. 1:**
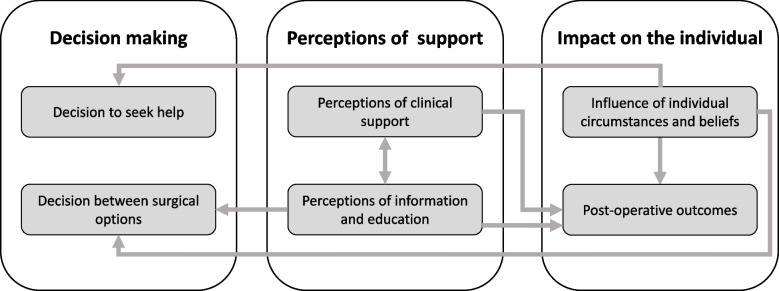
**Thematic map**. Thematic map of the three themes (bold text, white background), six subthemes (plain text, grey background) and conceptual links between the subthemes (grey arrows)

Three themes, each with two subthemes, were identified: decision-making, perceptions of support and impact on the individual. Fig. 1 provides a thematic map representing the conceptual links between the subthemes. Our PPI contributors recognised their own experiences in the themes, particularly regarding the factors that influenced their decision-making and inadequacies in the information provided by health professionals.

### Theme 1: decision-making

#### Decision to seek help

Participants described the signs/symptoms that influenced their decision to seek help for their ankle conditions. All participants sought help when their ankle pain affected their ability to carry out valued activities, such as sleeping and caring responsibilities. One participant described the emotional impact of the pain:

“Well as I say, as soon as I put that foot on the floor it was brrr, the pain was terrific. In fact, I got to such a point where, well I just couldn’t cope with it, could I?” (Rose, 85, TAR).

Impact on walking was described by four participants. One participant found it difficult to walk on uneven ground or for long distances. Others found it difficult to fully weight-bear, describing needing crutches and limping with a stick. Reduced mobility negatively affected participants’ engagement with their usual activities. One participant described being housebound and relying on others for transport. In addition, a change in his foot’s shape contributed to one participant’s decision to seek help. This participant described his foot as being *“tipped over”* and wearing down the outside of the shoe.

Participants’ views of ankle conditions also underpinned their decision to seek help, perceiving that their condition would have continued to progress without surgery:

“I would have been in a wheelchair full stop. I mean it was getting, even with crutches; it was you know, it was quite hard work.” (Jack, 74, post-TAR revision).

Three participants felt that their history of ankle injuries or sporting activities had led to a deterioration of their condition.

#### Decision between surgical options

Several factors influenced participants’ decision-making between TAR and ankle fusion. These included their surgeon’s advice/opinion, the participants’ perceptions of TAR/ankle fusion and their individual circumstances. Whilst one participant’s surgeon expressed no preference between TAR and ankle fusion, two participants were advised to undergo ankle fusion to avoid further surgery. Other participants were only offered ankle fusion surgery. It was unclear if this was their surgeon’s decision based on the participant’s clinical presentation, or the only procedure provided by the service.

Some participants perceived TAR to be a much bigger operation than ankle fusion. Prior to a post-operative discussion with his surgeon, one participant believed ankle fusion was *“a routine thing”*. Some participants associated ankle fusion with a permanently fixed ankle and a subsequent negative change in gait/appearance, such as having a limp or dragging walk, and being lopsided, as opposed to having a normal gait with a TAR:

“I’m not an old king that’s going to be hobbling about, thank you.” (Edward, 72, TAR, awaiting revision).

TARs were considered to provide joint flexibility, which participants felt would enable them to do more activities. Correspondingly, one participant felt lucky to have had a TAR even though it failed. Two participants identified *“vanity”* as a factor in their decision-making as they thought that fusion was more likely to lead to a limp. One of these participants felt that undergoing TAR would give her a *“proper ankle”*, aligning with another participant’s perception of TAR as *“getting it done properly”*:

“With the fusion you’re not as mobile as you want to be. Well put it this way, you’re not as mobile as I want to be okay. I wouldn’t be able to do the things I do now if I had a fused ankle. So that was my motivation to getting it done properly.” (Henry, 74, TAR).

For one participant, desire to reduce pain and improve deformity influenced his decision to proceed with ankle fusion, with no concerns of the ankle being fixed.

Age also influenced participants’ decision-making. For example, two participants requiring revision revealed they had originally chosen TAR because they were younger (approximately 60), linking this to being more active and not wanting to limp at a younger age. Both participants perceived that ankle fusion was more acceptable now they were older (in their 70s). One participant favoured ankle fusion due to his age and the potential concerns about having more surgery when he was older:

“Because I don’t want to go another five years and find that I’ve got to be in plaster for another you know, six months or whatever.” (George, 71, fusion).

Conversely, an 85-year-old participant reported that she wanted a TAR regardless of her age.

### Theme 2: perceptions of support

#### Perceptions of information and education

Pre-operative education was provided or sought through surgeon consultations, discussions with previous patients, and the Internet. Whilst one participant was completely happy with the pre-operative education provided, most felt that education was lacking on topics such as what to expect pre- and post-operatively, medication side effects, recovery time and the practical issue of not being able to climb stairs:

“I didn’t know how I was going to be. I didn’t realise, it may sound foolish, but I had no idea that I wouldn’t be able to climb the stairs even.” (Rose, 85, TAR).

Two participants reported they were not provided with sufficient written information:

“The actual information, I think is more general orthopaedic surgery rather than sort of specific to your ankle. And I think it would be better if there was something more about the fusion. I did find more because I went on the Internet and had a look.” (Sarah, 77, fusion).

One participant appreciated their surgeon providing information through diagrams. Another felt her surgeon went through her radiographs in great detail and was excellent at communicating the results.

One participant had benefited from discussing ankle surgery with a previous patient, whereas another highlighted a lack of opportunities to receive personal insights from previous patients. Three participants sought further information about their procedure online, with one reflecting that not all online information is trustworthy and highlighting that online information had contributed to his unrealistic expectations of surgery:

“Unfortunately I had read on the Internet of people in America, after three weeks being out of plaster and running around and playing squash.” (George, 71, fusion).

#### Perceptions of clinical support

The majority of participants experienced a long waiting time for their procedure, with many needing cortisone injections and additional analgesic medications. One participant found the long wait for surgery upsetting. Four were transferred to different surgeons. A fifth actively sought a different surgeon due to his initial surgeon not performing TAR. The experience of being transferred between surgeons was perceived negatively by two participants, who felt forgotten and considered their care was disjointed through poor communication. In contrast, one participant had a short wait for surgery and attributed this to being transferred to a new surgeon with no waiting list. Only one participant confirmed they received physiotherapy pre-operatively.

Although participants were generally satisfied with the surgical procedure itself, some participants felt they were discharged from hospital too soon, with one revealing an overall feeling of abandonment post-operatively:

“I felt that I was, I came out too soon, it was all a muddle. So basically, that is my only, my real complaint, was that there was not enough information and I was sent out too soon.” (Rose, 85, TAR).

Whilst some participants felt the wait for post-operative outpatient physiotherapy was too long, the physiotherapy itself was perceived as helpful and reassuring. Participants also described a range of other post-operative support mechanisms including district nurses, mobility aids, home modifications, pain medication, footwear and orthotic devices.

### Theme 3: impact on the individual

#### Influence of individual circumstances and beliefs

In addition to influencing their decision-making, individual circumstances influenced participants’ perceptions of their post-operative outcomes. Some participants compared their ankle surgery to previous surgeries, whilst others compared their outcomes to those of their peers. Participants’ comorbidities also had an influence:

“It’s just that I, I am quite a good healer and I was quite disappointed that I hadn’t healed so quickly. […] Yeah, after I came out of the boot, I had phenomenal heel pain; it was just like walking on glass. And you know what that was, plantar fasciitis, but I didn’t know that at the time. And I got very, very worried that the fusion had not worked.” (George, 71, fusion).

During their recovery, four participants relied on family support to cope with the impact of their operation. Most participants believed self-motivation was important for recovery, for example through exercising and *“getting the ankle stronger”.* One participant also recognised the impact of ankle surgery in relation to occupation, commenting on being able to work in the building trade for 10 years after TAR with very few problems despite doing *“heavy work”.*

#### Post-operative outcomes

Participants reported some initial negative signs and symptoms following surgery, such as pain, swelling, difficulty performing activities of daily living and changes in sensation:

“I would just step into the shower this morning, I thought you daft bag, you haven’t took your socks off. And actually, I had, but that’s how that foot felt.” (Mary, 66, post TAR revision).

In the longer term, most participants were pleased with the outcome of their surgery overall, identifying how it had improved their pain and mobility. One participant thought that the surgery was successful because the surgeon was happy, despite ongoing symptoms. Another participant was pleased with his outcome initially and felt it was unfortunate that the joint replacement failed and additional surgery was required.

In contrast, one participant revealed she was unhappy, stating that she would not have it done again even though her surgeon was happy with the outcome of her TAR revision and her ankle appeared aligned radiographically. This participant felt that her ankle did not look completely straight, she *“wasn’t walking right”* and she had ongoing swelling, pains and mobility issues. Another participant similarly stated that, despite her fusion surgery resolving her resting pain, it was still a struggle and painful to walk.

## Discussion

By exploring patients’ lived experiences across the TAR/ankle fusion pathway, this study has helped to address an important gap in existing literature. The findings highlight an array of factors that may contribute to patients’ decision to undergo TAR or ankle fusion and affect how they perceive their care and outcomes. Some factors were common to all participants in our study. For example, pain affecting their valued activities was central to all participants’ decision to seek help. Other factors, such as the influence of participants’ comorbidities on their post-operative outcomes, reflected participants’ individual circumstances and beliefs. A key finding was that the education and clinical support participants received had an important impact on their experiences and perceptions. Despite this, most participants felt that their education and support needs were not fully met.

Our findings about patients’ decision-making, both in terms of when to seek help and which type of surgery to have, largely align with those of a qualitative study by Zaidi et al. [7]. As in this study, Zaidi et al. [7] identified that patients may obtain information about TAR and ankle fusion from health professionals and peers, with surgeons having a particularly key influence. Our study expands these findings by highlighting that surgeons and peers may also affect patients’ perceptions of their post-operative outcomes. Another novel finding identified in our study is that some patients may prefer TAR due to *“vanity”*, believing that TAR but not ankle fusion will enable them to avoid limping. Correspondingly, our findings suggest that patients may believe TAR will provide them with a *“proper ankle”* and be concerned about the lack of joint flexibility following ankle fusion. This corresponds with a qualitative study by Conlin et al. [8], in which patients who had undergone both TAR and ankle fusion felt that their TAR was more like a *“normal ankle”* (p.1155), whilst their ankle fusion provided a feeling of stability.

Our findings highlight that the perception that TAR offers better gait/activity outcomes may result in patients believing TAR is more appropriate at a younger age. This contrasts with the perceptions of surgeons identified in a recent survey by Tai et al. [19], which suggested that surgeons believe ankle fusion is more appropriate for younger patients. This may be because younger patients are likely to live longer and have higher activity levels, potentially causing more rapid TAR wear and increasing the chances of failure [20]. Conversely, a recently published retrospective cohort study found that the risk of minor and major revision post-TAR did not differ significantly between younger and older patients [21]. A potential contributory factor to these differing findings is that perceptions/definitions of a *‘younger’* patient vary. For example, two participants in our study perceived themselves as relatively young at 60 years old, whereas Tai et al. [19] referred to younger patients as those less than 40 years old.

Our finding that the education patients receive can substantially affect their experiences and perceptions corresponds with literature on other orthopaedic procedures. This suggests that education may influence a patient’s knowledge, skills, expectations, mental wellbeing and satisfaction [22–24]. Our findings demonstrate that pre- and post-operative education prior to TAR or ankle fusion was often insufficient and there was variability in the type of information provided. Written information has been shown to facilitate understanding among patients awaiting foot and ankle surgery [25] but not all participants in our study were provided with sufficient written information. Some participants sought further information about their surgical procedure from the Internet, similar to those in previous studies [7, 9]. The readability and quality of online information about ankle surgery is often poor [26], highlighting the important role of health professionals in challenging any misconceptions and providing evidence-based education throughout the care pathway.

Participants in our study perceived various limitations in the clinical support they received such as disjointed care, being discharged too soon, and having a long wait for post-operative physiotherapy. In addition, we found limited evidence of input from podiatrists, orthotists, or occupational therapists, despite the potential for these allied health professionals to support patients both pre- and post-operatively. Whilst most participants in our study appeared satisfied with their surgery overall, two participants who had undergone fusion expressed dissatisfaction due to ongoing discomfort and difficulty walking. Correspondingly, a prospective cohort study by Younger et al. [27] found patients were more likely to report improved satisfaction with their symptoms post-TAR than post-fusion, although the absolute satisfaction scores were similar for both procedures. Other studies have also suggested that patients’ satisfaction levels with TAR and ankle fusion are similar [5, 28].

### Limitations

Our findings must be viewed in light of this study’s limitations. Whilst our sample size of seven is in line with typical sample sizes for phenomenological studies [11, 13], interviewing further participants may have provided additional insights. One option to help address this would have been to continue interviewing participants until we reached data saturation. However, the concept of saturation is inconsistently defined, challenging to assess and arguably inappropriate for the reflexive thematic analysis approach we employed [29]. An alternative to saturation is the concept of information power proposed by Malterud et al. [30]. This suggests that the more relevant information that can be obtained from a sample to address the study aim, the fewer participants are necessary. The seven interviews we conducted provided detailed and relevant information that directly addressed our study aim, supporting the appropriateness of our sample size.

Overall, our purposive sampling strategy captured a broad range of patients regarding gender, age, type of surgery, and duration since surgery. However, we did not interview any patients who had undergone ankle fusion revision surgery. In addition, we recruited participants from a single orthopaedic centre; therefore, our findings may not be transferable to other patients due to variations in service provision. We excluded patients unable to understand and speak English to ensure that participants could provide true informed consent and participate fully in the interviews. In addition, we did not record the ethnicity of participants to help preserve their anonymity, as relatively low numbers of patients undergo TAR. This meant we could not explore the impact of differing cultures and ethnicities on patients’ perceptions. This is an important limitation as previous research has highlighted racial disparities in utilisation of TAR and ankle fusion [31, 32]. Another potential limitation is that the participants were aware that members of the research team were based at the recruitment site, which may have discouraged them from criticising their care. This did not appear to be a substantial issue as the participants expressed both positive and negative perceptions.

### Implications for clinical practice and future research

Our findings demonstrate that education and clinical support for patients undergoing TAR/ankle fusion is not always perceived as adequate by patients. Identifying and addressing such inadequacies is vital to inform patients’ decision-making, guide their preparations for surgery and support their post-operative recovery. We found that patients’ individual circumstances and beliefs had a strong influence on their decision-making and perceptions of their post-operative outcomes, highlighting the need to personally tailor support. Given the lack of definitive evidence in this area, developing consensus-based guidelines on pre- and post-operative support for patients undergoing TAR/ankle fusion would be valuable to guide service provision. There is also a need for further qualitative research to address areas not covered in our study. For example, further research is required to explore which outcomes are most important to patients undergoing TAR/ankle fusion and other stakeholders to inform the development of a standardised core outcome set for ankle surgery.

## Conclusions

This study has provided insights into patients’ perceptions of TAR and ankle fusion surgery by exploring patients’ lived experiences across the entire TAR/ankle fusion care pathway. Numerous factors that may influence patients’ decision-making and perceptions of their care and outcomes were identified. Some of these were common to all participants, whilst others reflected participants’ individual circumstances and beliefs. The education and clinical support participants received had a substantial impact on their experiences and perceptions, yet appeared to be inadequate. These findings emphasise the importance of providing patients with adequate education about TAR and ankle fusion, and tailoring pre- and post-operative education and clinical support to the individual patient.

## Supplementary Information

**Additional File 1.** “I could have a proper ankle” – a qualitative study of patients’ perceptions of total ankle replacement and ankle fusion surgery: Interview topic guide. Topic guide used to guide each semi-structured interview.

## Data Availability

The datasets generated and/or analysed during the current study are not publicly available to ensure that participants’ anonymity is maintained.

## Abbreviations

OA: osteoarthritis
TAR: total ankle replacement
UK: United Kingdom
