# Supplementary material for: “I could have a proper ankle” – a qualitative study of patients’ perceptions of total ankle replacement and ankle fusion surgery
Source: J Foot Ankle Res. 2022 Dec 12;15:88. doi: 10.1186/s13047-022-00595-8 (PMC9743489; doi:10.1186/s13047-022-00595-8)
Supplement: Supplementary file 1 — Additional File 1. “I could have a proper ankle” – a qualitative study of patients’ perceptions of total ankle replacement and ankle fusion surgery: Interview topic guide. Topic guide used to guide each semi-structured interview. [file 13047_2022_595_MOESM1_ESM.pdf]

“I could have a proper ankle” – a qualitative study of patients’ perceptions of total ankle replacement and ankle fusion surgery: Interview topic guide

Ankle replacement version

Pre-interview introduction

- ☐ Explain the purpose of the interview and confidentiality.
- ☐ Confirm the participant has read the patient information sheet and consent form.
- ☐ Ask if the participant has any questions about the patient information sheet and consent form.
- ☐ Confirm the participant gives permission for the interview to be recorded using a digital voice recorder. Explain that a transcript will be produced from the recording and that all references or names that might identify the participant will be removed.
- ☐ Complete and collect the consent form.

Interview prompts

1. Could you tell me a bit about what led you to having an ankle replacement?

2. How did you reach the decision to have an ankle replacement?

3. What were you hoping having an ankle replacement would achieve?

4. Could you tell me a bit about the information you received <u>before</u> your ankle replacement?

5. Could you tell me a bit about the information you received <u>after</u> your ankle replacement?

6. Did you receive any treatment <u>before</u> your ankle replacement?

7. Did you receive treatment <u>after</u> your ankle replacement?

Participants who have not had/are not awaiting revision surgery

8. Do you think your ankle replacement was successful?

Participant awaiting revision

9. Could you tell me a bit about why you have decided to have revision surgery?

Participant who has had revision surgery

10. a) Could you tell me a bit about why you decided to have revision surgery?
    b) Do you think your revision surgery was successful?

11. Is there anything else you would like to mention that we have not already discussed?

Interview closure

'I would like to thank you for taking the time to talk with me. Your interview has been really helpful and interesting. I would just like to re-assure you again that we will keep all of the information you have shared confidential.'

Ankle fusion version

Pre-interview introduction

- ☐ Explain the purpose of the interview and confidentiality.
- ☐ Confirm the participant has read the patient information sheet and consent form.
- ☐ Ask if the participant has any questions about the patient information sheet and consent form.
- ☐ Confirm the participant gives permission for the interview to be recorded using a digital voice recorder. Explain that a transcript will be produced from the recording and that all references or names that might identify the participant will be removed.
- ☐ Complete and collect the consent form.

Interview prompts

1. Could you tell me a bit about what led you to having an ankle fusion?

2. How did you reach the decision to have an ankle fusion?

3. What were you hoping having an ankle fusion would achieve?

4. Could you tell me a bit about the information you received <u>before</u> your ankle fusion?

5. Could you tell me a bit about the information you received <u>after</u> your ankle fusion?

6. Did you receive any treatment <u>before</u> your ankle fusion?

7. Did you receive treatment <u>after</u> your ankle fusion?

8. Do you think your ankle fusion was successful?

9. Is there anything else you would like to mention that we have not already discussed?

Interview closure

‘I would like to thank you for taking the time to talk with me. Your interview has been really helpful and interesting. I would just like to re-assure you again that we will keep all of the information you have shared confidential.’
